# Supplementary material for: Auto-regulation of Rab5 GEF activity in Rabex5 by allosteric structural changes, catalytic core dynamics and ubiquitin binding
Source: eLife. 2019 Nov 13;8:e46302. doi: 10.7554/eLife.46302 (PMC6855807; doi:10.7554/eLife.46302)
Supplement: Supplementary file 1. [file elife-46302-supp1.docx]

|  |  |  |  | Top scoring model | | Model with max XL satisfied | | Total XL satisfied in cluster | |
| --- | --- | --- | --- | --- | --- | --- | --- | --- | --- |
|  | 4-HB, Vps9 Rotation | RpBD Positioning | #Cluster | Satisfied XL | Fraction | Satisfied XL | Fraction | Satisfied XL | Fraction |
| *4q9u* | rigid | rigid | #Cluster 0 | 106 | 0.87 | 107 | 0.88 | 107 | 0.88 |
| *4q9u* | rigid | rigid | #Cluster 1 | 105 | 0.86 | 107 | 0.88 | 107 | 0.88 |
| *4q9u* | rigid | rigid | #Cluster 2 | 104 | 0.85 | 107 | 0.88 | 107 | 0.88 |
| *4q9u* | flexible | rigid | #Cluster 0 | 114 | 0.93 | 116 | 0.95 | 120 | 0.98 |
| *4q9u* | flexible | rigid | #Cluster 1 | 113 | 0.93 | 119 | 0.98 | 121 | 0.99 |
| *4q9u* | flexible | rigid | #Cluster 2 | 112 | 0.92 | 116 | 0.95 | 119 | 0.98 |
| *4q9u* | rigid | flexible | #Cluster 0 | 107 | 0.88 | 109 | 0.89 | 115 | 0.94 |
| *4q9u* | rigid | flexible | #Cluster 1 | 108 | 0.89 | 109 | 0.89 | 116 | 0.95 |
| *4q9u* | rigid | flexible | #Cluster 2 | 103 | 0.84 | 106 | 0.87 | 107 | 0.88 |
| *4q9u* | flexible | flexible | #Cluster 0 | 116 | 0.95 | 119 | 0.98 | 121 | 0.99 |
| *4q9u* | flexible | flexible | #Cluster 1 | 115 | 0.94 | 119 | 0.98 | 121 | 0.99 |
| *4q9u* | flexible | flexible | #Cluster 2 | 118 | 0.97 | 119 | 0.98 | 120 | 0.98 |
| *4n3z* | rigid | rigid | #Cluster 0 | 109 | 0.89 | 110 | 0.90 | 112 | 0.92 |
| *4n3z* | rigid | rigid | #Cluster 1 | 109 | 0.89 | 110 | 0.90 | 111 | 0.91 |
| *4n3z* | rigid | rigid | #Cluster 2 | 108 | 0.89 | 111 | 0.91 | 111 | 0.91 |
| *4n3z* | flexible | rigid | #Cluster 0 | 116 | 0.95 | 117 | 0.96 | 118 | 0.97 |
| *4n3z* | flexible | rigid | #Cluster 1 | 116 | 0.95 | 117 | 0.96 | 118 | 0.97 |
| *4n3z* | flexible | rigid | #Cluster 2 | 114 | 0.93 | 117 | 0.96 | 117 | 0.96 |
| *4n3z* | rigid | flexible | #Cluster 0 | 108 | 0.89 | 109 | 0.89 | 109 | 0.89 |
| *4n3z* | rigid | flexible | #Cluster 1 | 107 | 0.88 | 109 | 0.89 | 109 | 0.89 |
| *4n3z* | rigid | flexible | #Cluster 2 | 107 | 0.88 | 109 | 0.89 | 110 | 0.90 |
| *4n3z* | flexible | flexible | #Cluster 0 | 115 | 0.94 | 116 | 0.95 | 121 | 0.99 |
| *4n3z* | flexible | flexible | #Cluster 1 | 111 | 0.91 | 117 | 0.96 | 119 | 0.98 |
| *4n3z* | flexible | flexible | #Cluster 2 | 114 | 0.93 | 117 | 0.96 | 118 | 0.97 |
