# Supplementary material for: Auto-regulation of Rab5 GEF activity in Rabex5 by allosteric structural changes, catalytic core dynamics and ubiquitin binding
Source: eLife. 2019 Nov 13;8:e46302. doi: 10.7554/eLife.46302 (PMC6855807; doi:10.7554/eLife.46302)
Supplement: Supplementary file 2. [file elife-46302-supp2.docx]

| Key Resources Table | | | | |
| --- | --- | --- | --- | --- |
| Reagent type (species) or resource | Designation | Source or reference | Identifiers | Additional information |
| cell line (*Homo-sapiens*) | Cervical (cancerous, Adult) | (Bucci, Parton et al., 1992, Villasenor, Nonaka et al., 2015) | HeLa Kyoto | STR and mycoplasma testing: ongoing |
| Rabex5 KO cell line (*Homo-sapiens*) | Cervical (cancerous, Adult) | In house, Materials and Methods: Rabex5 Knockout and Rescue | HeLa Kyoto Rabex5 Knockout | STR and mycoplasma testing: ongoing |
| transfected  construct (*Homo sapiens*) | hRabex5 | Synthesized by GenScript | pCMV5 Flag-Smad1 | BglII and SalI sites used for ligation |
| transfected  construct (*Homo sapiens*) | hRabexΔLinker | Synthesized by GenScript | pCMV5 Flag-Smad1 | BglII and SalI sites used for ligation |
| transfected  construct (*Homo sapiens*) | hRabexΔUBD | Synthesized by GenScript | pCMV5 Flag-Smad1 | BglII and SalI sites used for ligation |
| antibody | anti-EEA1 (Rabbit polyclonal) | (Franke, Repnik et al., 2019) |  | IF (1:1000), WB (1:1000) |
| antibody | anti-HA-tag (Mouse monoclonal) | Sigma |  | IF (1:200) |
| recombinant DNA reagent | pOEM-1 N-His | Oxford Expression Technologies, This paper | Rabex5 | NotI and AscI sites used for ligation |
| recombinant DNA reagent | pOEM-1 N-His | Oxford Expression Technologies, This paper | Rabex5ΔRpBD | NotI and AscI sites used for ligation |
| recombinant DNA reagent | pOEM-1 N-His | Oxford Expression Technologies, This paper | Rabex5ΔUBD | NotI and AscI sites used for ligation |
| recombinant DNA reagent | pOEM-1 N-His | Oxford Expression Technologies, This paper | Rabex5ΔLinker | NotI and AscI sites used for ligation |
| recombinant DNA reagent | pOEM-1 N-His | Oxford Expression Technologies, This paper | RabexCAT | NotI and AscI sites used for ligation |
| recombinant DNA reagent | pOEM-1 N-GST | Oxford Expression Technologies, This paper | Rabaptin5 | NotI and AscI sites used for ligation |
| software | HDX Workbench | Omics Informatics |  |  |
| chemical compound | GTPgS | Sigma | [G8634](https://www.sigmaaldrich.com/catalog/product/sigma/g8634?lang=de&region=DE) |  |
| chemical compound | GTP | Sigma | [10106399001](https://www.sigmaaldrich.com/catalog/product/roche/10106399001?lang=de&region=DE) |  |
| chemical compound | Mant-GDP | Jena Biosciences | NU-204L |  |
| chemical compound | Urea | Sigma | [U4883](https://www.sigmaaldrich.com/catalog/product/sigma/u4883?lang=de&region=DE) |  |
| chemical compound | deuterium oxide | Sigma | [151882](https://www.sigmaaldrich.com/catalog/product/aldrich/151882?lang=de&region=DE) |  |
| chemical compound | TFA | Sigma | [T6508](https://www.sigmaaldrich.com/catalog/product/sigald/t6508?lang=de&region=DE) |  |
| Rabex_cr1 | RNA guide |  | TCTTAACAGCTTGAAGATAA |  |
| Rabex_cr2 | RNA guide |  | ATCTTCAAGCTGTTAAGAGA |  |
| Rabex_cr3 | RNA guide |  | AGTGAAAAAATGTATTTAGA |  |
| Rabex_cr4 | RNA guide |  | GACGTACCCACTGCAGGGCT |  |

Bucci C, Parton RG, Mather IH, Stunnenberg H, Simons K, Hoflack B, Zerial M (1992) The small GTPase rab5 functions as a regulatory factor in the early endocytic pathway. Cell 70: 715-28

Franke C, Repnik U, Segeletz S, Brouilly N, Kalaidzidis Y, Verbavatz JM, Zerial M (2019) Correlative single-molecule localization microscopy and electron tomography reveals endosome nanoscale domains. Traffic 20: 601-617

Villasenor R, Nonaka H, Del Conte-Zerial P, Kalaidzidis Y, Zerial M (2015) Regulation of EGFR signal transduction by analogue-to-digital conversion in endosomes. Elife 4
